# Supplementary material for: Population Genomics of the Critically Endangered Brazilian Merganser
Source: Animals (Basel). 2023 Dec 6;13(24):3759. doi: 10.3390/ani13243759 (PMC10741106; doi:10.3390/ani13243759)
Supplement: Supplementary file 1 [file animals-13-03759-s001.zip › animals-2722023-supplementary.pdf]

Population Genomics of the Critically Endangered Brazilian Merganser

Davidson P. Campos [1], Henry Paul Granger-Neto [1], José E. Santos Júnior [1], Pierre Faux [2] and Fabrício R. Santos [1,*]

[1] Department of Genetics, Ecology and Evolution, Instituto de Ciências Biológicas, Universidade Federal de Minas Gerais, Belo Horizonte 31270-901, MG, Brazil; davidsonpcampos@gmail.com (D.P.C.); hp.granger98@gmail.com (H.P.G.-N.); jrsantos140782@yahoo.com.br (J.E.S.J.)
[2] GenPhySE, Université de Toulouse, INRAE, ENVT, 31326 Castanet-Tolosan, France; pierre.faux@inrae.fr
* Correspondence: fsantos@icb.ufmg.br

Supporting information

Table S1: List of samples used in this work and their respective occurrence areas, and geographic coordinates (lat/long).

Samples	Occurrence Area	Lat	Long
JAL202	Jalapão	-10.630692	-46.75354
JAL203	Jalapão	-10.630692	-46.75354
JAL204	Jalapão	-10.630692	-46.75354
JAL205	Jalapão	-10.630692	-46.75354
VEA066	Veadeiros	-14.28388889	-47.7975
VEA069	Veadeiros	-14.28388889	-47.7975
VEA070	Veadeiros	-14.28388889	-47.7975
VEA071	Veadeiros	-14.28388889	-47.7975
PAR074	Paranaíba	-19.07055556	-46.94333333
PAR180	Paranaíba	-19.074486	-46.938445
PAR206	Paranaíba	-18.662724	-47.066072
PAR207	Paranaíba	-18.662724	-47.066072
PAR208	Paranaíba	-18.662724	-47.066072
PAR209	Paranaíba	-18.662724	-47.066072
PAR210	Paranaíba	-19.074486	-46.938445
CAN018	Canastra	-20.3225	-46.47222222
CAN023	Canastra	-20.31555556	-46.52944444
CAN035	Canastra	-20.32166667	-46.50472222
CAN038	Canastra	-20.32166667	-46.50472222
CAN098	Canastra	-20.321654	-46.504038
CAN099	Canastra	-20.321654	-46.504038
CAN100	Canastra	-20.321654	-46.504038
CAN147	Canastra	-20.334125	-46.468472
CAN148	Canastra	-20.323391	-46.501575
CAN151	Canastra	-20.252688	-46.622608
CAN155	Canastra	-20.252688	-46.622608
CAN159	Canastra	-20.252688	-46.622608
CAN198	Canastra	-20.352173	-46.347669
CAN200	Canastra	-20.352173	-46.347669
CAN211	Canastra	-20.321654	-46.504038

Table S2: Pairwise kinship between sequenced individuals.

*Values of kinship between 0.0442 and 0.0884 are in the theoretical range of 3rd-degree relatives, values between 0.0884 and 0.177 are 2nd-degree relatives, and values between 0.177 and 0.354 are 1st-degree relatives. Values above 0.354 are monozygotic twins, or more likely in the case of birds, the same individuals.

	CAN018	VEA069	VEA070	PAR210	PAR206	JAL203	PAR207	JAL202	JAL204	CAN200	CAN147	CAN211	CAN035	JAL205	PAR209	VEA071	PAR208	PAR074	PAR180	CAN098	CAN155	CAN148	CAN038	VEA066	CAN100	CAN159	CAN099	CAN023	CAN151	CAN198
**CAN018**	0.5000	-	-	-	-	-	-	-	-	-	-	-	-	-	-	-	-	-	-	-	-	-	-	-	-	-	-	-	-	-
**VEA069**	-0.3256	0.5000	-	-	-	-	-	-	-	-	-	-	-	-	-	-	-	-	-	-	-	-	-	-	-	-	-	-	-	-
**VEA070**	-0.1212	0.0538	0.5000	-	-	-	-	-	-	-	-	-	-	-	-	-	-	-	-	-	-	-	-	-	-	-	-	-	-	-
**PAR210**	-0.1055	-0.2489	-0.0745	0.5000	-	-	-	-	-	-	-	-	-	-	-	-	-	-	-	-	-	-	-	-	-	-	-	-	-	-
**PAR206**	-0.3631	-0.5780	-0.2625	-0.1990	0.5000	-	-	-	-	-	-	-	-	-	-	-	-	-	-	-	-	-	-	-	-	-	-	-	-	-
**JAL203**	-0.2033	-0.2737	-0.0143	-0.1304	-0.2639	0.5000	-	-	-	-	-	-	-	-	-	-	-	-	-	-	-	-	-	-	-	-	-	-	-	-
**PAR207**	-0.2617	-0.4234	-0.1908	-0.1156	0.2075	-0.1593	0.5000	-	-	-	-	-	-	-	-	-	-	-	-	-	-	-	-	-	-	-	-	-	-	-
**JAL202**	-0.3064	-0.3187	-0.0277	-0.2203	-0.4148	0.2426	-0.2824	0.5000	-	-	-	-	-	-	-	-	-	-	-	-	-	-	-	-	-	-	-	-	-	-
**JAL204**	-0.1956	-0.2337	0.0086	-0.0931	-0.2943	0.2674	-0.1962	0.2540	0.5000	-	-	-	-	-	-	-	-	-	-	-	-	-	-	-	-	-	-	-	-	-
**CAN200**	-0.0154	-0.1813	0.0136	-0.0392	-0.1659	-0.0672	-0.0689	-0.1198	-0.0442	0.5000	-	-	-	-	-	-	-	-	-	-	-	-	-	-	-	-	-	-	-	-
**CAN147**	-0.0549	-0.2125	-0.0306	-0.0451	-0.1091	-0.1017	-0.0501	-0.1677	-0.0829	0.0967	0.5000	-	-	-	-	-	-	-	-	-	-	-	-	-	-	-	-	-	-	-
**CAN211**	-0.0661	-0.2500	0.0052	-0.1053	-0.1934	-0.0693	-0.1274	-0.1155	-0.0380	0.1842	0.0263	0.5000	-	-	-	-	-	-	-	-	-	-	-	-	-	-	-	-	-	-
**CAN035**	-0.0633	-0.1387	0.0169	-0.0136	-0.1806	-0.1145	-0.0892	-0.1706	-0.0916	0.0672	0.0907	-0.0075	0.5000	-	-	-	-	-	-	-	-	-	-	-	-	-	-	-	-	-
**JAL205**	-0.2291	-0.1795	0.0477	-0.1198	-0.2916	0.2865	-0.1632	0.0252	0.2897	-0.0512	-0.0786	-0.0414	-0.0982	0.5000	-	-	-	-	-	-	-	-	-	-	-	-	-	-	-	-
**PAR209**	-0.3614	-0.5267	-0.2355	-0.1749	0.1133	-0.2120	0.1990	-0.3502	-0.2171	-0.1171	-0.1063	-0.2112	-0.1509	-0.2326	0.5000	-	-	-	-	-	-	-	-	-	-	-	-	-	-	-
**VEA071**	-0.2735	-0.0403	0.1347	-0.1776	-0.3527	-0.1230	-0.2383	-0.1585	-0.0918	-0.0668	-0.1113	-0.1147	-0.0498	-0.0595	-0.3230	0.5000	-	-	-	-	-	-	-	-	-	-	-	-	-	-
**PAR208**	-0.1966	-0.3043	-0.1138	-0.0557	0.1940	-0.1109	0.2982	-0.2620	-0.1520	-0.0396	-0.0059	-0.0593	-0.0197	-0.1448	0.2129	-0.1875	0.5000	-	-	-	-	-	-	-	-	-	-	-	-	-
**PAR074**	-0.0564	-0.1461	0.0185	0.1163	-0.0448	-0.0285	0.0289	-0.0927	-0.0164	0.0921	0.0469	0.0688	0.0682	-0.0054	-0.0278	-0.0679	0.0920	0.5000	-	-	-	-	-	-	-	-	-	-	-	-
**PAR180**	0.0076	-0.0776	0.0408	0.1661	0.0078	0.0185	0.0524	-0.0522	0.0227	0.0788	0.0756	0.0710	0.0816	0.0370	0.0131	-0.0147	0.0950	0.1911	0.5000	-	-	-	-	-	-	-	-	-	-	-
**CAN098**	-0.0774	-0.2138	-0.0085	-0.0611	-0.1350	-0.0676	-0.0924	-0.1285	-0.0556	0.0385	0.0257	0.0265	0	-0.0441	-0.1441	-0.1157	-0.0458	0.0472	0.1179	0.5000	-	-	-	-	-	-	-	-	-	-
**CAN155**	-0.0549	-0.2125	-0.0306	-0.0451	-0.1091	-0.1017	-0.0501	-0.1677	-0.0829	0.0967	0.5000	0.0263	0.0907	-0.0786	-0.1063	-0.1113	-0.0059	0.0469	0.0756	0.0257	0.5000	-	-	-	-	-	-	-	-	-
**CAN148**	-0.0239	-0.1852	0.0135	-0.0484	-0.1816	-0.0797	-0.0825	-0.1322	-0.0492	0.2653	0.0939	0.2212	0.0610	-0.0470	-0.1392	-0.0862	-0.0333	0.0839	0.1178	0.0472	0.0939	0.5000	-	-	-	-	-	-	-	-
**CAN038**	-0.0449	-0.1915	-0.0484	0.006	-0.2349	-0.1468	-0.1215	-0.2220	-0.1201	0.0409	0.0316	-0.0556	0.2625	-0.1285	-0.1729	-0.0983	-0.0566	0.0533	0.0997	-0.0411	0.0316	0.0368	0.5000	-	-	-	-	-	-	-
**VEA066**	-0.1581	-0.0696	0.1100	-0.1111	-0.2811	-0.0058	-0.2093	-0.0505	0.0019	-0.0258	-0.0683	-0.0837	-0.0532	0.0314	-0.2901	0.0563	-0.1743	-0.0511	0.0146	-0.0334	-0.0683	-0.0438	-0.0752	0.5000	-	-	-	-	-	-
**CAN100**	-0.0952	-0.1953	0	-0.0685	-0.1221	-0.0828	-0.0667	-0.1458	-0.0794	0.0225	0.0150	0.0058	0.0205	-0.0807	-0.1208	-0.0819	-0.0163	0.0963	0.1184	0.2542	0.0150	0.0426	-0.0057	-0.0587	0.5000	-	-	-	-	-
**CAN159**	-0.0907	-0.2950	-0.0362	-0.1435	-0.2005	-0.1155	-0.1332	-0.1677	-0.0809	0.2188	0.0156	0.2560	0.0058	-0.0685	-0.2353	-0.1471	-0.1045	0.0347	0.0324	-0.0039	0.0156	-0.0483	-0.0737	-0.1364	-0.0522	0.5000	-	-	-	-
**CAN099**	-0.0244	-0.0947	0.0360	0.0020	-0.1609	-0.0756	-0.0823	-0.1129	-0.0316	0.0368	0.0552	0.0095	0.0678	-0.0369	-0.1425	-0.0562	-0.026	0.0874	0.1378	0.2537	0.0552	0.0710	0.0507	0.0074	0.2571	-0.0434	0.5000	-	-	-
**CAN023**	-0.0181	-0.2179	-0.0139	-0.0060	-0.1332	-0.0923	-0.1049	-0.1288	-0.0282	0.0616	0.0653	0.0327	0.0334	-0.0617	-0.1626	-0.0664	-0.0385	0.0904	0.1066	0.0600	0.0653	0.0517	0	-0.0415	0.0230	0.0300	0.0584	0.5000	-	-
**CAN151**	0.1797	-0.2976	-0.0966	-0.0933	-0.2447	-0.1236	-0.1885	-0.2086	-0.0890	0.0143	-0.0041	0.0360	-0.0183	-0.1047	-0.2668	-0.2067	-0.1124	0.0081	0.0374	0.0062	-0.0041	-0.0162	-0.0293	-0.1183	-0.0274	0.0376	0.0021	0.1303	0.5000	-
**CAN198**	-0.1045	-0.2646	-0.0560	-0.1345	-0.1552	-0.1376	-0.1303	-0.1692	-0.0923	0.1459	0	0.0602	0.0193	-0.0897	-0.1944	-0.1782	-0.1104	0.0365	0.0476	-0.0215	0	-0.0212	-0.0873	-0.1181	-0.0300	0.2092	-0.0098	0.0857	0.0286	0.5000

Table S3: Individual inbreeding coefficients (F). INDV = individual; O(HOM) = observed number of SNPs in homozygosity; O(HET) = observed number of SNPs in heterozygosity. The expected homozygosity was 659.7, the expected heterozygosity was 263.3 and the number of analyzed SNPs was 923.

INDV	O(HOM)	O(HET)	F
JAL202	696	227	0.13776
JAL203	676	247	0.06179
JAL204	654	269	-0.02177
JAL205	650	273	-0.03696
VEA066	655	268	-0.01797
VEA069	717	206	0.21753
VEA070	609	314	-0.19270
VEA071	658	265	-0.00658
PAR074	643	280	-0.06355
PAR180	575	348	-0.32184
PAR206	757	166	0.36947
PAR207	718	205	0.22133
PAR208	692	231	0.12257
PAR209	736	187	0.28970
PAR210	687	236	0.10358
CAN018	742	181	0.31249
CAN023	661	262	0.00482
CAN035	646	277	-0.05216
CAN038	659	264	-0.00278
CAN098	652	271	-0.02937
CAN099	654	269	-0.02177
CAN100	663	260	0.01242
CAN147	643	280	-0.06355
CAN148	709	214	0.18714
CAN151	649	274	-0.04076
CAN155	685	238	0.09598
CAN159	683	240	0.08838
CAN198	649	274	-0.04076
CAN200	665	258	0.02001
CAN211	664	259	0.01621

Table S4: Analysis of Molecular Variance for different groupings of *Mergus octosetaceus* populations: Serra da Canastra National Park (Canastra); Jalapão State Park (Jalapão); Chapada dos Veadeiros National Park (Veadeiros); Alto Paranaíba region (Paranaíba).

	Run	Population groupings	Percentage of variation within populations	F_{ST}	F_{SC}	F_{CT}
All individuals	1	Canastra+Paranaíba X Veadeiros X Jalapão	81.53	0.1847	0.0979	0.0962
	2	Canastra+Paranaíba X Veadeiros+Jalapão	82.51	0.1749	0.1261	0.0559
	3	Canastra X Paranaíba X Veadeiros+Jalapão	84.63	0.1538	0.1877	-0.0418
Only unrelated individuals	1	Canastra+Paranaíba X Veadeiros X Jalapão	86.71	0.1329	0.0627	0.0750
	2	Canastra+Paranaíba X Veadeiros+Jalapão	86.63	0.1337	0.0666	0.0718
	3	Canastra X Paranaíba X Veadeiros+Jalapão	89.10	0.1090	0.0818	0.0296
